# Alsidium Blodgettii in Consumption and Scrofulous Diseases

**Published:** 1856-10

**Authors:** Benjamin Palmer

**Affiliations:** Pittsfield, Mass.


*Alsidium Blodgettii in Consumption and Scrofulous Diseases.*

EDITOR NEW YORK MEDICAL TIMES:—

*Dear Sir*—I wish to direct the attention of the Medical profession to a marine plant, discovered by Dr. A. E. Rue, on the coast of Australia. Dr. R. is very confident that he has discovered some very valuable medicinal properties in this plant, and states, in the most positive terms, that it is a specific in consumption and scrofulous diseases.

I have every confidence in Dr. Rue as not being disposed to speculate on the Materia Medica, or attract attention by new and fashionable therapeutic agents; and through his kindness I have been furnished at different times with the medicine as prepared and used by him, and also a specimen of the plant. Upon examination, I find the same plant was originally discovered by Dr. Blodgett, and is accurately and minutely described and classified by Prof. Wm. H. Harvey, of Dublin University, in his classification of Algæ. The following is his description of it:—"Alsidium Blodgettli—frond sub-compressed below, terete above, decompound pinnate; pinnæ alternate, patent, close, virgate, the lowest very long, set with short setaceous, spinous-toothed, alternate, distichous ramuli; upper branches short and sub-simple; conceptacle pedicellate, inflated, urceolate, variously placed on the ramuli."

I have used this medicine with the most gratifying results in many cases in my own practice where there was every sign of *tubercular deposition*, some of which were in quite an advanced stage; yet, not satisfied with my own experience, I placed it in the hands of a few medical friends, who were equally well pleased with its success in these diseases. From what I consider the duty of every medical man, I have decided to make these facts known to the profession, hoping that any additional facts pertaining to the history or medicinal properties of the plant will be reported. Yours, etc.,

BENJAMIN PALMER, M.D.

PITTSFIELD, MASS., July, 1856.

